# Programmed DNA breaks in lymphoid cells: repair mechanisms and consequences in human disease

**DOI:** 10.1111/imm.12547

**Published:** 2015-11-18

**Authors:** Jana Prochazkova, Joanna I. Loizou

**Affiliations:** ^1^ CeMM Research Centre for Molecular Medicine of the Austrian Academy of SciencesViennaAustria

**Keywords:** class switch recombination, DNA repair, immunodeficiency, non‐homologous end‐joining, V(D)J recombination, cancer

## Abstract

In recent years, several novel congenital human disorders have been described with defects in lymphoid B‐cell and T‐cell functions that arise due to mutations in known and/or novel components of DNA repair and damage response pathways. Examples include impaired DNA double‐strand break repair, as well as compromised DNA damage‐induced signal transduction, including phosphorylation and ubiquitination. These disorders reinforce the importance of genome stability pathways in the development of lymphoid cells in humans. Furthermore, these conditions inform our knowledge of the biology of the mechanisms of genome stability and in some cases may provide potential routes to help exploit these pathways therapeutically. Here we review the mechanisms that repair programmed DNA lesions that occur during B‐cell and T‐cell development, as well as human diseases that arise through defects in these pathways.

**Abbreviations**
AID: activation induced deaminase
AT: ataxia telangiectasia
ATM: ataxia telangiectasia mutated
ATLD: ataxia telangiectasia‐like disorder
BCR: B‐cell receptor
CSR: class switch recombination
DSB: DNA double‐strand break
MRN: complex comprising the exonuclease MRE11, RAD50 and NBS1
NBS: Nijmegen breakage syndrome
NHEJ: non‐homologous end‐joining
DNA‐PKcs: DNA‐dependent protein kinase catalytic subunit
RIDDLE: radiosensitivity, immunodeficiency, dysmorphic features and learning difficulties
RSS: recombination signal sequences
SCID: severe combined immunodeficiency
SSB: DNA single‐strand break
ssDNA: single‐stranded DNA
TCR: T‐cell receptor
UNG: uracil‐DNA glycosylase
V(D)J: variable, diversity, joining

## Introduction

DNA double‐strand breaks (DSB) are the most toxic form of DNA damage. However, such breaks are also generated in a programmed manner in mammalian lymphoid cells. B‐cell receptor (BCR) and T‐cell receptor (TCR) loci consist of variable (V), diversity (D) and joining (J) genes that are recombined together in a process called V(D)J recombination. This is a mechanism that generates a wide repertoire of B and T cells, which enables these cells to recognize an almost unlimited number of different antigens. The process of V(D)J recombination is tightly controlled and the DSBs must be repaired correctly to avoid the persistence of deleterious DNA lesions or resulting translocations. Mature B cells undergo two further genomic alterations: class switch recombination (CSR) and somatic hypermutation. The major DNA repair pathway responsible for repairing DNA lesions during V(D)J recombination and CSR is non‐homologous end‐joining (NHEJ). Defects in proteins that function in this pathway lead to a failure to repair programmed DNA breaks correctly. This has severe consequences for affected individuals, ranging from immune deficiency and neurological defects, to a predisposition to the development of malignancies. The aim of this review is to summarize the current knowledge of the NHEJ pathway that functions in the resolution of programmed DNA breaks and the consequences of mutations within this pathway for human health, in the cellular compartment of B and T cells. For an overview of the literature on somatic hypermutation, we direct readers to the following reviews.^1^, ^2^

## Mechanisms of recombination in lymphoid cells

V(D)J recombination and CSR of programmed DSBs in lymphocytes is tightly regulated on multiple levels, starting with initiation of recombination and ending with proper joining of the free DNA ends. Here, we discuss the steps involved in this process and the factors involved, within the context of B and T cells.

### V(D)J recombination

**Figure 1:**
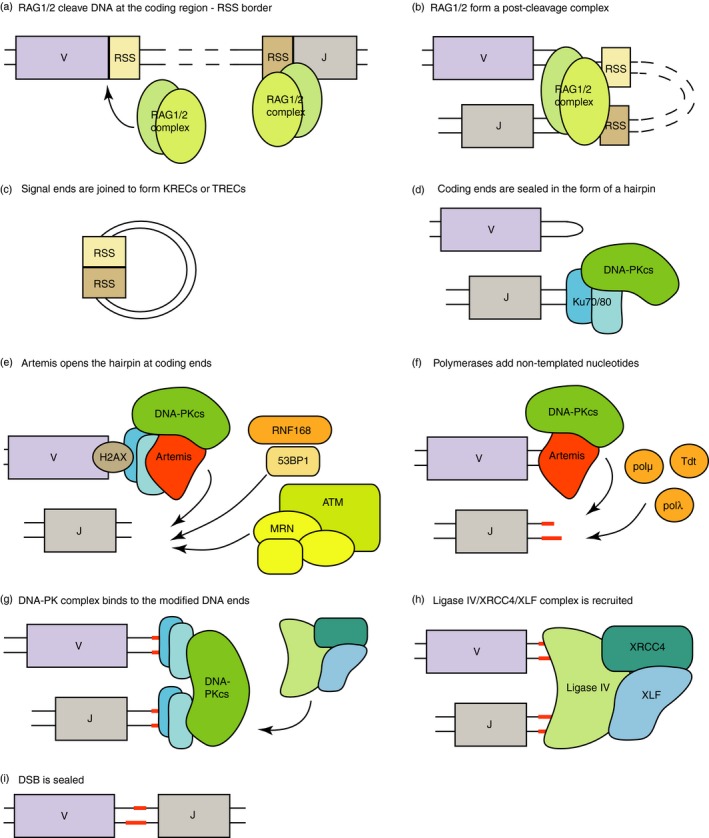
Schematic representation of the various stages and proteins involved in the generation and repair of programmed DNA lesions during V(D)J recombination that occur in B and T cells.

In germ‐line cells, each of the numerous V, D and J genes has a coding region that is flanked by recombination signal sequences (RSS). V(D)J recombination is initiated by recombination activating genes (RAG1 and RAG2). These enzymes are sequence‐specific nucleases that recognize and cleave the RSS to generate a synapsis complex (Fig. 1a, b). The DSBs at the RSS ends are blunt and are immediately joined by NHEJ to form K‐deleting recombination excision circles in B cells or TCR excision circles in T cells, that mark cells that have recently undergone V(D)J recombination (Fig. 1c). The DSB that is formed at the coding end of the locus is sealed in the form of a hairpin, which is subsequently opened by the endonuclease Artemis and joined with another coding end by the NHEJ machinery (Fig. 1d–i). As RAG1 and RAG2 are expressed only transiently in specific developmental stages in lymphoid cells, the recombination process is tightly regulated.^3^ Furthermore, recombination is also controlled by accessibility of the chromatin structure for the given locus, which is mediated by histone acetylation and methylation.^4^

The generation and repair of programmed V(D)J DNA breaks in B and T cells are restricted to the G0/G1 phase of the cell cycle. Here, DNA lesions are processed by NHEJ.^5^ Following the generation of the DSB by RAG1/2, the coding end of the BCR or TCR gene locus is sealed with a hairpin (Fig. 1d). The hairpin is bound by Ku70 and Ku80, which function as a heterodimer along with the DNA‐dependent protein kinase catalytic subunit (DNA‐PKcs; a PIKK kinase) (Fig. 1d). As Ku70/Ku80 and DNA‐PKcs modulate RAG‐mediated cleavage^6^ by assisting the selection of proper RSS combination, this implies that the DNA–PK complex might be involved in the recombination process upstream of RAG cleavage. Indeed, in mice Ku70 and/or Ku80 are absolutely essential for V(D)J recombination and their deletion leads to severe combined immunodeficiency (SCID).^7^, ^8^ As of yet, *Ku70/80* mutations have not been identified in patients. Next DNA‐PKcs phosphorylates Artemis and in doing so activates Artemis to open the hairpin and therefore enable the progression of V(D)J recombination^9^ (Fig. 1e).

Downstream DNA repair proteins next accumulate at the break site in response to accumulation of phosphorylated H2AX. The histone variant is phosphorylated by a PIKK kinase (DNA‐PKcs and also ATM), upon which it is known as *γ*H2AX. ATM (for ataxia telangiectasia mutated) is a serine/threonine protein kinase that functions in the repair of DNA lesions. It acts in concert with the MRN complex (comprising the exonuclease MRE11, RAD50 and NBS1, which is formed stably only when all three proteins are present).^10^, ^11^ Whereas DNA‐PKcs contributes to signalling DSBs at signal ends, the kinase activity of ATM, which belongs within the same family of kinases as DNA‐PKcs, can partially compensate for its function at signal ends.^12^, ^13^

As the ATM substrate p53 localizes at V(D)J break sites, it has been suggested that ATM provides surveillance at the site of breaks and helps to suppress potential oncogenic translocations when repair fails.^11^ Hence, ATM maintains DNA ends in repair complexes generated during V(D)J recombination (Fig. 1e) and therefore ATM deficiency leads to instability of post‐cleavage complexes and loss of coding ends from these complexes.^14^ The MRN complex associates with ATM after induction of DNA damage and is required for ATM activation and recruitment of ATM to DSBs.^15^ During V(D)J recombination, MRN deficiency leads to the aberrant joining of RAG‐induced DSBs and to the accumulation of unrepaired coding ends, so establishing a functional role for MRN in the repair of RAG‐mediated DNA DSBs. Moreover, these defects in V(D)J recombination are remarkably similar to those observed in ATM‐deficient lymphocytes, suggesting that ATM and MRN function in the same DNA DSB response pathways during lymphocyte antigen receptor gene assembly.^16^

Ubiquitination additionally plays an important role in the accumulation of repair proteins to sites of DNA damage, which enables efficient signalling after programmed DSBs. The chromatin‐associated E3 ubiquitin ligase RNF168 co‐localizes with NHEJ proteins such as 53BP1, and increases the local ubiquitination of proteins, hence retaining them at the site of damage and facilitating downstream signalling cascades^17^, ^18^ (Fig. 1e).

Before joining, DNA ends can be modified by terminal deoxynucleotidyl transferase, which can insert random nucleotides (Fig. 1f). This mechanism generates even greater diversity of the BCR and TCR. Indeed, lack of terminal deoxynucleotidyl transferase leads to a restricted B‐cell and T‐cell repertoire.^19^ Similarly, polymerases *μ* and *λ* can perform nucleotide fill‐in synthesis and also template‐independent synthesis, hence generating microhomologies, as well as direct and inverted repeats, all of which occur at V(D)J junctions^20^ (Fig. 1f).

The DNA–PK complex recruits the core NHEJ ligation machinery consisting of DNA ligase IV, XRCC4 and XLF (Fig. 1h). Both XRCC4 and XLF bind to ligase IV, which is responsible for sealing the loose DNA ends. Recently, a 3′ exonuclease with single‐stranded DNA (ssDNA) endonuclease activity named polynucleotide kinase and aprataxin‐like forkhead‐associated protein (PALF) was implicated in NHEJ. It was shown to resect 3′ overhanging nucleotides and permit XRCC4‐DNA ligase IV to complete the joining process in a manner that is as efficient as Artemis; however, it is not able to open hairpins. Reduction of PALF *in vivo* reduces the joining of incompatible DNA ends.^21^ Recently, PAXX (paralogue of XRCC4 and XLF) was identified as a new XRCC4 family member and component of the NHEJ pathway. It is recruited to DNA damage sites where it interacts with Ku dimers and mediates DNA repair by assembling the core NHEJ proteins and mediating DNA ligation.^22^

### Class switch recombination

B cells that have undergone V(D)J recombination can additionally undergo CSR where one set of *IgH* constant regions is replaced with another, hence allowing B cells to secrete different antibody classes. The initiating enzyme responsible for generating the DSBs in CSR is activation induced deaminase (AID). AID belongs to the APOBEC family of DNA cytidine deaminases. AID activity is tightly regulated transcriptionally, post‐transcriptionally and post‐translationally by various factors^23^ and functions on transcriptionally active switch regions. Following the deamination of cytidine to uracil the base excision repair pathway removes uracil by uracil‐DNA glycosylase (UNG). The phosphodiester bond at the abasic site is subsequently cleaved by the apurinic/apyrimidinic endonuclease, leaving a DNA single‐stranded break (SSB). Since the deoxyribose phosphate group is still attached to the 5′ end of the lesion, the lyase activity of DNA polymerase *β* removes this group before processing of the lesion. The close proximity of DNA SSBs on both strands of DNA act as precursors to DSBs. Alternatively, mismatch repair can convert distal SSBs to DSBs.

The resulting DSBs are processed by NHEJ, largely as described above. It was thought that because hairpins are not generated in CSR, Artemis does not play a role in this process.^24^ Therefore the role of the DNA–PK complex in CSR was considered to be in regulating end processing, possibly by phosphorylating other proteins.^25^ However, it has since been shown that Artemis is indeed involved in CSR, where its nuclease activity may function in resolving more complex lesions that are generated during CSR.^26^ The polymerases involved in CSR are thought to include the error prone polymerases, polymerase *η* and REV1.^27^, ^28^

## Genes and diseases associated with defective recombination in B and T cells

Mutations in several genes that code for key players in the generation and repair of programmed DNA double‐strand breaks in B and T cells are associated with human immune deficiencies. However, for many such disorders the aetiology remains unclear. In recent years, new genes have been described to function in the NHEJ pathway and at the same time, new mutations have been characterized that are causative for B‐cell and T‐cell immune deficiencies. These are discussed following and summarised in Table 1.

**Table 1:** Human disorders associated with defects in V(D)J recombination and class switch recombination that occur due to inefficient generation or repair of programmed DNA lesions

Gene name	Associated human disease/phenotype
*RAG1/2*	Severe combined immunodeficiency (SCID); Omenn syndrome; malignancy (infrequent)
*AICDA*	Hyper‐IgM syndrome type 2
*UNG*	Hyper‐IgM syndrome type 5
*Artemis*	Radiosensitive SCID; Omenn syndrome; leaky SCID; radiation sensitivity; malignancy
*PRKDC*	Radiosensitive SCID; microcephaly, neurological defects; radiation sensitivity; autoimmunity
*ATM*	Ataxia telangiectasia; immunodeficiency; lung infections; radiation sensitivity; malignancy
*MRE11*	Ataxia telangiectasia‐like disorder; lung infections; radiation sensitivity; malignancy
*NBN*	Nijmegen breakage syndrome; microcephaly; immunodeficiency; radiation sensitivity; malignancy
*RAD50*	Phenotype similar to Nijmegen breakage syndrome (NBS); microcephaly; chromosomal instability
*RNF168*	Riddle syndrome; immunodeficiency; radiation sensitivity
*DNA ligase IV*	Phenotype similar to NBS and/or radiosensitive SCID; microcephaly; immunodeficiency; malignancy
*XRCC4*	Microcephaly; facial dysmorphism
*XLF*	Microcephaly; immunodeficiency; developmental delay; radiation sensitivity

### RAG1/2 deficiency

Mutations in *RAG1* and/or *RAG2* which lead to a complete loss of function of the gene, result in SCID.^29^ More commonly, enzymatic activity of the mutated *RAG* genes is partially retained and as such, patients present with a wide range of clinical outcomes. *RAG1* and *RAG2* mutations were identified in patients with symptoms ranging from a severe hindrance of the immune system to autoimmune defects affecting different organs. Hypomorphic mutations of *RAG1* and *RAG2* manifest as an Omenn syndrome, an autosomal recessive form of SCID accompanied by erythrodermia, hepatosplenomegaly, lymphadenopathy, eosinophilia, increased serum IgE levels and alopecia. In this disease, T and B cells are almost completely absent because of the inability to form antigen receptors via V(D)J recombination.^29^ The remaining T cells are largely oligoclonal, activated and infiltrate the skin and intestine. Recently, a further *RAG1* hypomorphic mutation was identified in a patient with common variable immunodeficiency‐like disease.^30^ The mutation led to a decrease in RAG1 activity by 50% and the patient presented with an autoimmune neutropenia but also liver granuloma and B‐cell lymphoma.

In addition, mutations affecting various RAG domains have been described, revealing more about the function of these enzymes. Mutations within the RAG1 domain that are crucial for interacting with RAG2 have been identified.^31^, ^32^ Moreover, it was recently shown that mutations affecting the recombination activity of RAG1 correlate with clinical outcome. Near complete loss of the enzymatic activity leads to SCID but, where enzymatic activity remains, phenotypes are less severe.^33^ These phenotypes include not only Omenn syndrome but also leaky SCID, SCID with expansion of *γδ* T cells (often associated with cytomegalovirus infections), combined immune deficiency with granuloma and/or autoimmunity and idiopathic CD4^+^ T‐cell lymphopenia. In addition, the group of van der Burg has shown that patients with a similar N‐terminal truncating RAG1 mutation have surprisingly different clinical outcomes.^34^ This study demonstrates that the type of *RAG1* mutation and the level of residual RAG1 recombinase activity are not the only determinants predicting the clinical phenotype. As suggested by the authors, this diversity also depends on a complex interplay between the often limited immune receptor repertoire, antigen or auto‐antigen exposure, the specificity of antigen receptors, and the timing and cell type involved in immune activation.^34^ Moreover, because RAG1 and RAG2 are also involved in formation of the post‐cleavage complex, mutation of *RAG1* rendering the enzyme proficient in DNA cleavage but deficient in post‐cleavage complex formation leads to impaired lymphocyte development and also to a predisposition to thymic lymphoma.^35^

### AID and UNG deficiency

Activation‐induced deaminase is the initiating enzyme that deaminates cytosine to uracil during the process of CSR. Therefore, loss of AID (encoded by the gene *AICDA*) leads to an inability for B cells to undergo CSR, which results in hyper‐IgM syndrome type 2 (HIGM2), a rare immunodeficiency characterized by normal or elevated serum IgM levels with absence of IgG, IgA and IgE, resulting in a profound susceptibility to bacterial infections. Ten independent mutations in the *AICDA* gene have been identified in 18 patients with HIGM2 from 12 families.^36^

Uracil‐DNA glycosylase functions immediately downstream of AID by removing uracil from DNA, and in doing so, promoting the generation of DNA lesions. Three patients with hyper‐IgM syndrome type 5 (HIGM5) were found to carry mutations in the *UNG* gene, all of which affected the catalytic domain of the UNG protein.^37^

### DNA‐PKcs deficiency

Mutations within *PRKDC*, the gene encoding DNA‐PKcs, lead to a radiosensitive SCID phenotype and currently a small number of patients with *PRKDC* mutations have been identified. One study has illustrated that even when the kinase activity of DNA‐PKcs and its DNA‐binding ability are preserved, the interaction with Artemis and the ability to activate this endonuclease are also required for successful V(D)J recombination.^38^ Moreover, some patients with a mutation in *PRKDC* not only manifest with the classical SCID symptoms but also suffer from growth failure and severe neurological defects, which indicates a requirement for DNA‐PKcs during human neuronal development.^39^ Interestingly, DNA‐PKcs was also shown to interact with autoimmune regulator (AIRE) to promote central T‐cell tolerance.^40^ Recently, two patients with *PRKDC* mutations were diagnosed with organ autoimmunity, suggesting an important role of DNA‐PKcs in the regulation of T‐cell tolerance.^41^

### Artemis deficiency

Mechanistically, Artemis forms a complex with DNA‐PKcs, which phosphorylates Artemis and so stimulates its endonuclease activity^9^ (Fig. 1e). Yet, the outcomes of *Artemis* and *PRKDC* mutations at the cellular level are different, suggesting additional functions for DNA‐PKcs in V(D)J recombination.^42^ Artemis inactivation causes radiosensitive T‐cell and B‐cell SCID in humans, that is characterized not only by the absence of T and B lymphocytes but also by cellular hypersensitivity to ionizing radiation due to the inability to efficiently repair DSBs.^43^ Most *Artemis* mutations identified localize in the N‐terminal region of the protein and manifest with loss of nuclease activity. Hypomorphic *Artemis* mutations with residual recombination and DNA repair activity were also identified. Such mutations manifest as combined immunodeficiencies of varying severity, such as leaky SCID.^44^ One such case was recently described in a patient with a disease mimicking hyper‐IgM syndrome with high serum IgM and low IgG and IgA levels, lymphocytosis and recurrent infections, intractable diarrhoea, growth retardation, systemic cytomegalovirus infection and sclerosing cholangitis. The patient also developed large granular lymphocytic leukaemia and died at a young age.^45^ In another case, a compound heterozygous mutation was identified in a patient whose clinical phenotype resembled Omenn syndrome with radiosensitivity.^46^ Moreover, two atypical cases of SCID have been reported in patients with splice site mutations, which resulted in low levels of *Artemis* transcript or low activity of the enzyme.^47^ This resulted in insufficient V(D)J recombination and a decrease in T‐cell and B‐cell compartments.

### RNF168 deficiency

Mutations in *RNF168* result in RIDDLE (radiosensitivity, immunodeficiency, dysmorphic features and learning difficulties) syndrome, which manifests with multiple symptoms including immune defects. Cells from the RIDDLE patient lack the ability to recruit the NHEJ‐promoting protein 53BP1 to sites of DSBs, where it localizes immediately after DNA damage and is involved in checkpoint control. Such cells exhibit hypersensitivity to ionizing radiation, cell cycle defects and impaired end‐joining during CSR in B cells.^48^, ^49^ A homozygous *RNF168* mutation was also reported in a patient with a syndrome mimicking ataxia telangiectasia (AT).^50^ Similar to a previous finding, patient cells displayed defective DNA repair and radiosensitivity due to defective ubiquitination of H2A and H2AX, leading to diminished recruitment of 53BP1 and BRCA1. In mice, loss of RNF168 causes immunodeficiency and radiosensitivity as well as increased genomic instability. Mechanistically, inactivation of RNF168 impairs long‐range V(D)J recombination in mouse thymocytes.^51^ A similar phenotype was shown in 53BP1‐deficient mice, which are radiosensitive and immunodeficient.^52^ The absence of 53BP1 in mice leads to impairment of distal V(D)J joining with extensive degradation of unrepaired coding ends and episomal signal joint re‐integration at V(D)J junctions. This results in apoptosis, loss of *TCRα* locus integrity and lymphopenia.^53^ However, others report that the defect of 53BP1‐deficient mice in V(D)J recombination is only mild^54^ and to date no human mutation in this gene has been reported.

### ATM and the MRN complex deficiency

Mutations leading to immune deficiencies have been described that are associated with most members of the ATM–MRN machinery. *ATM* mutations are the genetic cause of AT. This is an autosomal recessive disorder characterized by progressive cerebellar degeneration and an increased incidence of cancer. Importantly, ATM deficiency leads to a defect in thymocyte maturation that is associated with decreased efficiency in V‐J rearrangement of the endogenous *TCRα* locus, accompanied by increased frequency of unresolved *TCR‐Jα* coding end breaks.^55^ It was also shown that residual ATM kinase activity correlates with the severity of immune defects.^56^

Hypomorphic mutations of *MRE11* cause radiosensitive ataxia telangiectasia‐like disorder (ATLD). This disease is characterized by neurodegeneration and cancer predisposition, in a similar way to AT.^57^ In most patients, *MRE11* mutations affect the ability of the MRN complex to activate ATM. This is because mutations in the N‐terminal region of the protein destabilize MRN interactions whereas the C‐terminal mutations decrease the abundance of MRN complex.^58^

*NBN* (which encodes for the protein NBS1) hypomorphic mutations lead to Nijmegen breakage syndrome (NBS), which manifests with facial malformations, microcephaly and symptoms similar to AT including radiosensitivity, immune deficiency and increased risk of cancer.^59^ In murine models, loss of NBS1 hinders T‐cell development at an early developmental stage.^60^ Mechanistically, NBS1 depletion compromises loading of the MRN complex to V(D)J‐generated DSBs and thereby affects DNA end resection.^60^

The last member of the MRN complex, *RAD50*, was found to be mutated in a patient with microcephaly and mental retardation, short stature and bird‐like face, a disease phenotype similar to NBS.^61^ The RAD50 protein in this patient is unstable. Cells from this patient are radiosensitive, fail to relocalize the MRN complex in response to DNA damage, cannot efficiently activate ATM and exhibit chromosomal instability. Surprisingly though, at 23 years of age, the patient has yet to develop lymphoid or other malignancy and does not suffer from infections or exhibit other immunodeficient phenotypes.^61^ In addition, sequencing of patients with IgA deficiency and common variable immunodeficiency revealed that a patient suffering from radiosensitivity carried a heterozygous mutation resulting in a premature stop codon in the *RAD50* gene.^62^

### DNA ligase IV and XRCC4 deficiency

DNA ligase IV is involved in sealing DNA ends during V(D)J recombination (Fig. 1h) and its ablation in human B cells was shown to enhance cellular sensitivity to ionizing radiation as well as an inability to recombine the V(D)J genes.^63^ It is found in a complex with another NHEJ factor, XRCC4, and their interaction is essential for proper function (Fig. 1h). Similar to ligase IV deficiency, XRCC4‐deficient cells are sensitive to ionizing radiation and incapable of V(D)J recombination. Mechanistically, it was shown that the central region of XRCC4 is necessary and sufficient for binding and stimulating ligase IV activity.^63^, ^64^, ^65^

*Ligase IV* mutation leading to severely reduced enzymatic activity was originally identified in a highly radiosensitive patient with leukaemia.^66^ The clinical phenotype of patients with *ligase IV* mutation generally strongly resembles NBS and/or radiosensitive SCID, and includes immunodeficiency, developmental and growth defects and pronounced radiosensitivity. *Ligase IV* mutations in these patients disrupt the ligase domain of the protein or impair the interaction between ligase IV and XRCC4.^64^ In most patients, this leads to severe ablation of T and B lymphocytes and cells derived from these patients have impaired V(D)J recombination. However, in some patients, ligase IV residual activity is sufficient for recombining *TCRα* and *TCRβ* regions.^67^ In summary, ligase IV deficiencies present with various phenotypes where the degree of immune deficiency and neurological defects is highly variable.

A mutation in *XRCC4* identified by exome sequencing suggested this gene as a candidate disease‐causing mutation in a patient with primordial dwarfism.^68^ Most recently, *XRCC4* mutations were identified within five families in patients with microcephalic primordial dwarfism.^69^ The alterations found, substantially decrease XRCC4 protein levels leading to reduced cellular ligase IV activity and ionizing radiation‐induced DNA double‐strand break repair defects. However, none of the patients show signs of immune deficiency so far.^69^ Moreover, another three patients from a consanguineous family and one unrelated patient with mutations in *XRCC4* were identified. Similarly, the clinical phenotype presented in these patients was characterized by severe microcephaly, facial dysmorphism and short stature, in the absence of a recognizable immunological phenotype.^70^ A homozygous mutation resulting in a premature stop codon and very low levels of *XRCC4* transcript was found in two patients with progressive neurological defects, confirming the importance of DNA repair and XRCC4 in the brain.^71^ Another mutation that destabilizes XRCC4 protein, leading to proteasome‐mediated degradation, was also identified recently.^72^ Intriguingly, patient cells are radiosensitive and display a severe DSB repair defect but the patient only manifests with neurological defects without immune deficiency.^72^ Exome sequencing of two siblings with microcephaly and gonadal failure, identified another mutation leading to an in‐frame deletion of 23 amino acids, so expanding the spectrum of *XRCC4* mutations.^73^

In addition, Aprataxin, involved in DNA single‐strand break repair, was recently shown to interact with XRCC4. Cells lacking Aprataxin show increased levels of DNA breaks and the human disease characterized by Aprataxin deficiency is associated with progressive cerebellar degeneration, ataxia and oculomotor apraxia. This condition resembles other human diseases caused by deficiency in NHEJ pathway proteins but so far, immune deficiency was not described in these patients.^74^, ^75^, ^76^

### *XLF* deficiency

The XRCC4‐like factor (XLF, also called Cernunnos) shares sequence homology and structure similarity with XRCC4.^77^ Indeed, cells depleted for XLF display increased radiosensitivity and a defect in NHEJ.^77^ In addition, XLF was suggested to have overlapping functions with H2AX and 53BP1 in the assembly of DSB response factors on chromatin during V(D)J recombination.^54^

Patients with an *XLF* mutation display growth retardation, microcephaly and immunodeficiency characterized by a profound T‐cell and B‐cell lymphocytopenia due to defects in V(D)J coding. An increased cellular sensitivity to ionizing radiation, defective V(D)J recombination and impaired DNA‐end ligation is documented in cells from these patients.^77^, ^78^, ^79^

## Mechanisms of cancer development

Defects in the NHEJ repair pathway lead to genomic instability and cancer. Indeed, many of the key players in this process have been implicated in human disease, where cancer predisposition often accompanies the immune system defects (Table 1). There are two reasons for this, first, defective V(D)J recombination leads to oncogenic translocations. These can result in acute lymphoblastic leukaemias that arise from developing lymphocytes and also in more mature B‐cell lymphomas. Furthermore, CSR‐associated DSBs give rise to *IgH* translocations in mature B‐cell lymphomas and multiple myeloma.^80^, ^81^ Second, immune deficiency hinders the ability of the immune system to recognize and remove cancer cells in a timely and efficiently manner. This process, called immune surveillance, might be a contributing factor in at least some of the malignancies that accompany immune deficiencies.^82^

Hypomorphic *Artemis* mutations not only result in combined immunodeficiency but some patients have an increased susceptibility to lymphoma formation.^83^ *Artemis* mutation combined with p53 deficiency in mice leads to development of progenitor B‐cell lymphomas with translocations in immunoglobulin genes.^84^, ^85^ In humans, *Artemis* mutations were recently identified in patients with diffuse large B‐cell lymphomas.^86^ Some studies also suggest that patients with hypomorphic *Artemis* mutations are predisposed to cancer, potentially due to the involvement of Artemis in DSB repair during the G2 phase of the cell cycle.^87^

Ataxia telangiectasia is a well‐studied human disease caused by *ATM* mutations and characterized not only by neurological symptoms and immune deficiency but also by cancer predisposition. Patients develop mostly, but not exclusively, leukaemia and lymphoma and it was shown that the onset largely depends on the residual activity of ATM in these patients.^88^ In ATM‐deficient mice, which mimic the AT phenotype within the immune system, the importance of ATM in preventing T‐cell lymphomas has been well described. It was shown that translocations involving the *TCRα/δ* locus are the most common chromosomal aberrations. However, these translocations and amplifications involve V(D)J recombination‐initiated breaks in the *TCRδ* locus, as opposed to the *TCRα* locus, and arise independently of the *Eα*.^89^ Moreover, ATM‐deficient murine thymocytes are perturbed in passing through the *β*‐ or *γδ*‐selection checkpoint, leading in part to the developmental failure of T cells. Some of the clones with random or non‐random chromosomal translocations involving the *TCRα/δ* locus are selected, accumulate and give rise to malignant transformation.^90^ Deletion of the *TCRδ* enhancer (*Eδ*), which initiates *TCRδ* rearrangement, significantly improves *αβ* T‐cell output and effectively prevents t(12;14) translocations in ATM‐deficient mice. These findings support the notion that the genomic instability associated with V(D)J recombination at the *TCRδ* locus is the molecular origin of both lymphocytopenia and the signature t(12;14) translocations associated with ATM deficiency.^91^ In addition to these findings, it was shown that ATM‐deficient lymphocytes also contain telomere‐deleted ends produced by failed end joining during V(D)J recombination.^92^

Patients with NBS suffer from immune defects as well as a predisposition to develop malignancies. A wide range of cancer types was reported to occur in patients with this syndrome, with B‐cell and T‐cell lymphomas being the most common.^93^ However, other types of cancer, such as ovarian and breast cancer, have been reported.^94^ Moreover, heterozygous carriers of *NBN* mutations were also observed to have an enhanced risk of cancer.^95^

Another member of the MRN complex, MRE11, was implicated in cancer predisposition in a number of patients. Mutations in this gene were connected to breast cancer,^96^ colorectal and endometrial cancer^97^ and breast and lymphoid cancer.^98^ This is not a surprising finding as the presence of all three MRN subunits is vital for the stability of the MRN complex, which activates ATM. For example, a mutation in the N‐terminal region of MRE11, which destabilizes MRN, was found in a patient with a childhood cancer.^58^ Additionally, a different *MRE11* mutation was identified that preserves its nuclease and DNA‐binding ability and the ability to form the MRN complex and activate ATM. However, this mutation causes an increased sensitivity to ionizing radiation, defective Chk1 signalling and meiotic failure and additionally leads to cancer development, possibly through inefficient RAD50 binding.^99^

Several cases of B‐cell leukaemia and lymphoma were identified in patients with mutations in the *ligase IV* gene.^67^, ^100^ Moreover, murine models with hypomorphic *ligase IV* mutations develop thymic lymphoma, which further indicates the importance of ligase IV in the prevention of malignant transformation.^101^ In addition, DNA ligase I, a ligase involved in nucleotide excision repair and base excision repair, has recently been implicated in backup NHEJ, where it was associated with promoting chromosomal translocations.^102^ Moreover, single‐nucleotide polymorphisms in the *DNA ligase I* gene were found in patients with chronic myeloid leukaemia^103^ and some mutations are connected to increased risk of lung cancer.^104^

So far, no human cancer has been associated with *XLF* mutations. However, cells with decreased expression of XLF display increased sensitivity to agents that perturb DNA replication. Under replication stress, these cells exhibit impaired DSB repair and increased accumulation of cells in G2/M phases of the cell cycle. Moreover, *XLF* mutated and down‐regulated cells display greater chromosomal instability, particularly at chromosomal fragile sites, under replication stress, which may indicate a potential mechanism of preventing cellular transformation.^105^

## Conclusions and remaining questions

Human lymphoid deficiencies encompass a wide variety of disorders with broad symptoms. The aetiology of these diseases is not completely known, however, the advent of exome sequencing has allowed for the advancement of mutation identification. Genes involved in the NHEJ DNA repair pathway, which facilitates repair of programmed DNA breaks, play a major role in B‐cell and T‐cell disorders. In recent years, there have been many new reports describing how proteins involved in the NHEJ pathway function and what their contribution is to suppressing human immune deficiencies and also cancer. However, some crucial NHEJ genes have not yet been implicated as causative of human disease. Therefore, understanding the role of genes that are involved in the repair of programmed DNA breaks in the immune system is imperative, as this would allow for better management and treatment of immune deficiencies in the future.

## Disclosures

The authors declare that they have no conflict of interest.
